# Supplementary material for: Yeast Chromatin Mutants Reveal Altered mtDNA Copy Number and Impaired Mitochondrial Membrane Potential
Source: J Fungi (Basel). 2023 Mar 7;9(3):329. doi: 10.3390/jof9030329 (PMC10058930; doi:10.3390/jof9030329)
Supplement: Supplementary file 1 [file jof-09-00329-s001.zip › jof-2208325-supplementary.pdf]

Journal of *Fungi* MDPI

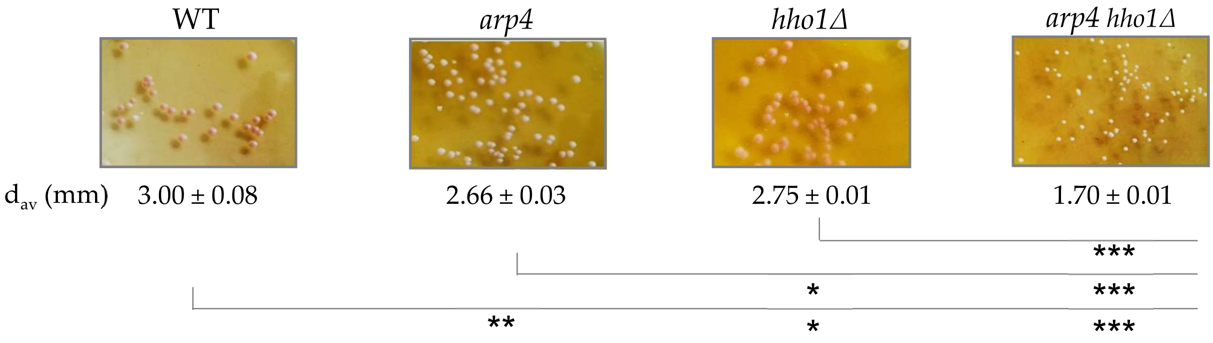

Figure S1. Colonies' size of the four studied strains**.** Cell suspensions from overnight cultures were appropriately diluted, plated on YPD plates and incubated for three days at 28°C to allow the formation of single colonies. The order from the smallest to the biggest d_{av} is *arp4 hho1Δ* << *arp4* ≈ *hho1Δ* < WT. Represented values are the mean measurements of two experiments; Statistical significance is designated with *p<0.05; **p<0.01; ***p<0.001

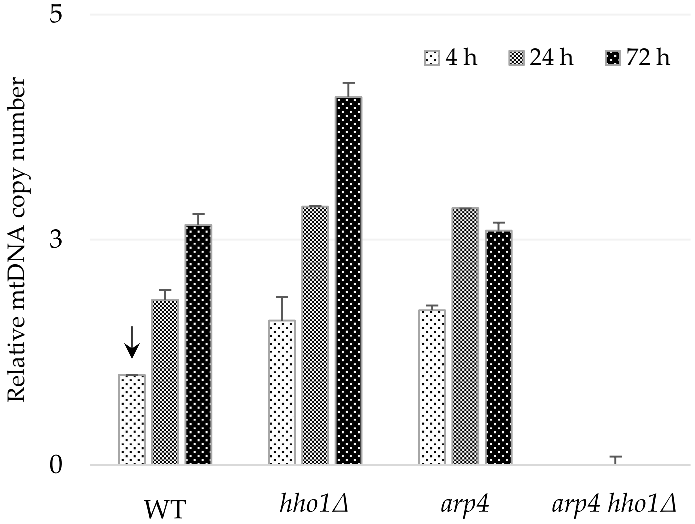

Figure S2. Relative mtDNA copy number in WT, *hho1Δ*, *arp4* and *arp4 hho1Δ* cells measured by real-time PCR with the primer pair 8F/8R of mt*COX3* sequence. The single-copy nuclear gene *ATG18* was used as a reference gene for normalization, and the sample WT 4th hour was applied to calibrate the results (an arrow indicates the calibrator). The primer pair 8F/8R is complimentary to the sequence of the *COX3* gene, and the amplified fragment is designated as "XIV" in Figure 3 and Table 2.

Table S1. Relative quantitation of *ATG18* and *CDC28* transcripts level by RT-qPCR. MEAN values are the average of two experiments ± SD.

	ATG18			*CDC28*		
	**4 h**	**24 h**	**72 h**	**4 h**	**24 h**	**72 h**
WT	10.3 ± 2.1	1.8 ± 0.7	1.2 ± 0.3	31.8 ±6.1	7.1 ± 3.9	1.4 ± 0.2
hho1Δ	3.8 ± 1.1	4.1 ± 1.8	2.1 ± 0.2	5.4 ± 2.9	2.6 ± 0.6	0.6 ± 0.3
arp4	15.1 ± 2.6	2.9 ± 1.3	1.5 ± 0.3	87.1 ± 72.7	2.8 ± 1.4	1.3 ± 0.3
arp4 hho1Δ	1.1 ± 0.5	1.0 ± 0.3	1.3 ± 0.4	2.3 ± 1.2	1.9 ± 0.2	1.5 ± 0.2
